# Long-term impacts of different posterior operations on curvature, neurological recovery and axial symptoms for multilevel cervical degenerative myelopathy

**DOI:** 10.1007/s00586-013-2741-5

**Published:** 2013-03-19

**Authors:** Wei Du, Linfeng Wang, Yong Shen, Yingze Zhang, Wenyuan Ding, Longxi Ren

**Affiliations:** 1 Department of Spine Surgery, The Third Hospital of Hebei Medical University, 139 Ziqiang Road, 050051 Shijiazhuang, China; 2 Department of Orthopaedic Surgery, Beijing Chuiyangliu Hospital, Beijing, China

**Keywords:** Multilevel cervical degenerative myelopathy, Posterior operations, Curvature index, Neurological recovery, Axial symptoms

## Abstract

**Purpose:**

To investigate the long-term impacts of different posterior operations on curvature, neurological improvement and axial symptoms for multilevel cervical degenerative myelopathy (CDM), and to study the relationship among loss of cervical lordosis, recovery rate and axial symptom severity.

**Methods:**

We retrospectively reviewed 98 patients with multilevel CDM who had undergone laminoplasty (Group LP, 36 patients), laminectomy (Group LC, 30 patients), or laminectomy with lateral mass screw fixation (Group LCS, 32 patients) between January 2000 and January 2005. Loss of curvature index (CI) was measured according to the preoperative and final follow-up radiographic parameters. The recovery rate was calculated based on the Japanese Orthopedic Association (JOA) score. Axial symptom severity was quantified by Neck Disability Index (NDI).

**Results:**

Analysis of final follow-up data showed significant differences among the three groups regarding loss of CI (*F* = 41.46, *P* < 0.001) between preoperative and final follow-up JOA scores (*P* < 0.001), final follow-up JOA score (*F* = 7.81, *P* < 0.001), recovery rate (*F* = 12.98, *P* < 0.001) and axial symptom severity (*χ* ^2^ = 18.04, *P* < 0.001). Loss of CI showed negative association with neurological recovery (*r* = −0.555, *P* < 0.001) and positive correlation with axial symptom severity (*r* = 0.696, *P* < 0.001).

**Conclusions:**

Excellent neurological improvement was obtained by LP and LCS for patients with multilevel CDM, while loss of CI in groups LP and LC caused a high incidence of axial symptoms. Loss of CI was correlated with poor neurological recovery and axial symptom severity. Lateral mass screw fixation can effectively prevent loss of postoperative cervical curvature and reduce incidence of axial symptoms.

## Introduction

Multilevel cervical degenerative myelopathy (CDM) is usually treated by different posterior decompression techniques. Laminoplasty has been considered as an effective and safe method to widen the spinal canal dimensions without removing the dorsal elements of the cervical spine [1, 2]. Laminectomy allows adequate decompression of the spinal cord, and can be performed safely and easily. Hence, a satisfactory surgical outcome in a short time is often seen in patients undergoing laminectomy [3]. However, some late operation-related complications have also been observed in laminoplasty and laminectomy, which include segmental instability, loss of cervical lordosis, neurological deterioration and axial symptoms [4–9]. Furthermore, several studies have shown that the remaining anterior compression might hinder the neurological recovery, if the segmental instability and kyphotic deformity were not corrected in the surgical management [4, 5, 7]. In recent years, laminectomy with lateral mass screw fixation, which can obtain adequate decompression of the spinal cord and immediate cervical stability, has been widely performed with favorable outcomes in the mid-term follow-up [9, 10]. It remains controversial whether different posterior operations have long-term adverse impacts on cervical curvature, neurological improvement and axial symptoms for multilevel CDM. Moreover, there are few reports on the relationship among curvature changes, recovery rate and axial symptom severity.

The purpose of this retrospective study was to investigate the long-term impacts of different posterior operations on curvature, neurological improvement and axial symptoms for the treatment of multilevel CDM. We also analyzed the relationship between loss of curvature index, recovery rate and axial symptom severity.

## Patients and methods

### Patient population

A total of 147 patients with multilevel CSM who had undergone different cervical posterior operations at our medical center were reviewed retrospectively from January 2000 to January 2005. Of the 147 patients, only 98 patients (68 men and 30 women) were eligible for final analysis in this study, while the rest 49 patients were excluded from the study because of the following reasons: suffering from diseases that had an adverse effect on the results (24 patients), radiological data incompletion (13 patients), loss to follow-up (10 patients) and death (2 patients). The patients’ age at the time of surgery ranged from 40 to 75 years (average 56.3 years), and the follow-up periods ranged from 7 to 12 years (average, 9.17 years). All data regarding age, gender, decompressed levels, preoperative symptoms and follow-up period were reviewed and statistically analyzed (Table 1).

**Table 1:** Patient characteristics

Characteristics	Group LP	Group LC	Group LCS
Total (*n*)	36	30	32
Mean age (years)	57.1 (42–75)	56.2 (43–74)	55.9 (40–72)
Gender			
Male	24	21	23
Female	12	9	9
No. of posterior decompressed levels			
C3	6	4	6
C4	9	8	8
C5	9	8	9
C6	8	7	7
C7	4	3	2
Presenting symptoms			
Weakness			
Upper extremity	22	16	19
Lower extremity	11	8	9
Extremity numbness hyperesthesia	19	15	16
Gait instability	21	17	18
Hyperreflexia	25	19	21
Hoffman sign	16	13	14
Babinski sign	9	6	7
Clonus	7	5	5
Follow-up time (year) (months)	9.2 (7.3–11.4)	9.4 (7.6–11.7)	8.9 (7.2–11.5)

Chi square test: no statistically significant differences among the three groups

Patients considered for the study had at least ≥3 levels of cervical spinal cord compression with accompanying symptoms and signs of cervical disk herniation, cervical spondylotic myelopathy, cervical spinal canal stenosis or segmental-type ossification of the posterior longitudinal ligament (OPLL). Exclusion criteria included cases with cervical trauma or continuous-type OPLL, cases with significant cervical anatomic deformity, active infection, and neoplasm, cases whose preoperative or final follow-up magnetic resonance imaging (MRI) and plain radiographs were not complete or interpretable because of motion/metal artifacts or poor quality, and the patients who had dropped out from the study during the follow-up periods.

All patients were classified into three groups based on the different posterior surgical managements. Patients in group LP consisted of 36 cases, with a mean age of 57.1 years (range 42–75 years) who underwent expansive open-door laminoplasty. Patients in group LC consisted of 30 patients, with a mean age of 56.2 years (range 43–74 years) who underwent laminectomy. Patients in group LC + Screw (LCS) included 32 patients, with a mean age of 55.9 years (range 40–72 years) who underwent laminectomy with lateral mass screw fixation. This study was approved by the Investigational Review Board at our institution, and informed consent was obtained from each patient.

### Surgical management

The open-door type of cervical en bloc laminoplasty described by Itoh and Tsuji [11, 12] was performed in group LP. One side of the lamina was opened, and the other side served as the hinge. Bone grafts from dissected spinous processes were put in the opened laminae and fixed with braided wires or nylon threads. Laminectomy was performed from pedicle to pedicle to ensure adequate spinal canal decompression in group LC. In group LCS, screws (Medtronic Sofamor Danek, Memphis, TN, USA) were placed bilaterally with the Magerl technique [13], rods of appropriate size were selected and bent to match the contour of the lateral masses and secured to the lateral masses by screws, and then laminectomy were performed based on the preoperative surgical planning. Numbers of posterior decompressed levels in each group were described detailedly in Table 1.

### Radiological assessments

**Fig. 1:**
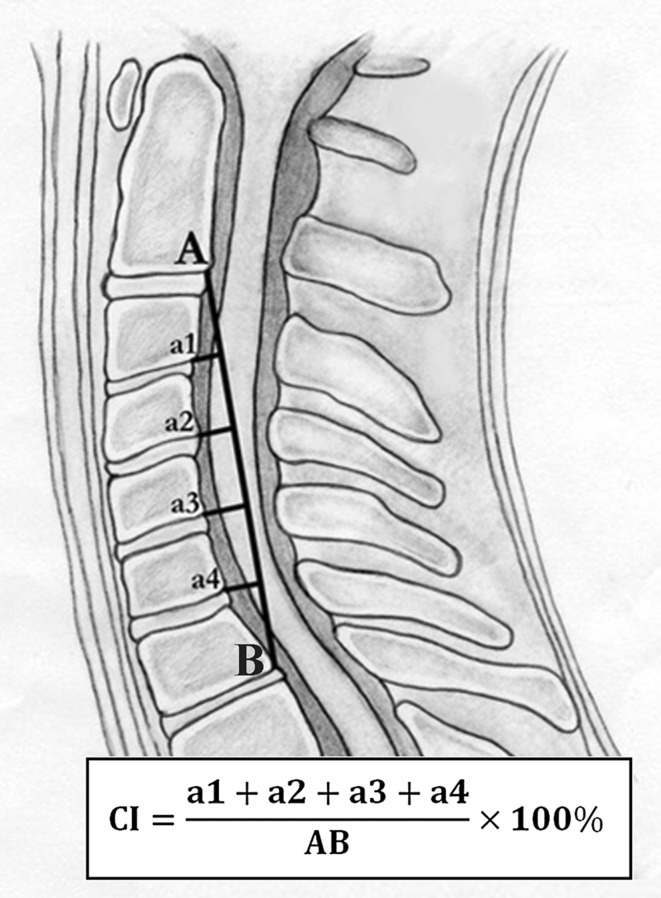
Calculation of the cervical curvature index (CI)

Preoperative and final follow-up cervical alignments were measured in the profile of neutral plain radiographs by curvature index (CI) as described by Ishihara [14] (Fig. 1).“a1” was defined as the distance from the posterior inferior edge of the C3 vertebral body to line “AB”, “a2, a3, and a4” using the same method. “AB” was defined as the distance from the posterior inferior edge of the C2 vertebral body to that of the C7 vertebral body.

Data measurements were performed three times with 200 % magnification for accuracy by the first and second authors independently, and the mean value was used for analysis. The intraobserver errors were less than 5 %.

### Clinical assessments

The neurological status of each patient was evaluated before surgery and at final follow-up according to the Japanese Orthopedic Association (JOA) disability scale. The neurological recovery rate was calculated using the Hirabayashi method [15]: (postoperative JOA score − preoperative score)/(17 − preoperative score) × 100 %. Recovery rates were graded as follows: ≥75 %, excellent; 50–74 %, good; 25–49 %, fair; and <25 %, poor.

The severity of preoperative and final follow-up axial symptoms in each group was quantified by Neck Disability Index (NDI, 0 = no disability, 50 = total disability) [16]. Subjects’ scores were calculated and ranked according to the standard NDI ranking system: 0–4, no disability; 5–14, mild disability; 15–24, moderate disability; 25–34, severe disability; >35, complete disability [17, 18].

### Statistical methods

All statistical analysis was performed using Statistical Analysis System software (version 9.13, SAS Institute Inc., USA). The Chi square test was applied for qualitative data. A paired *t* test was used to assess statistical significance of changes between final follow-up and preoperative parameters in each group. Statistical comparisons among the three groups were performed in loss of CI, the final follow-up JOA score and the neurological recovery rate using ANOVA and Student–Newman–Keuls test, and in the severity of axial symptoms using Kruskal–Wallis nonparametric ANOVA and Bonferroni *t* test. Pearson’s correlation coefficient was used to check the correlation among loss of curvature index, recovery rate and axial pain severity. A value of *P* < 0.05 was considered to be statistically significant.

## Results

### Radiographic results

**Fig. 2:**
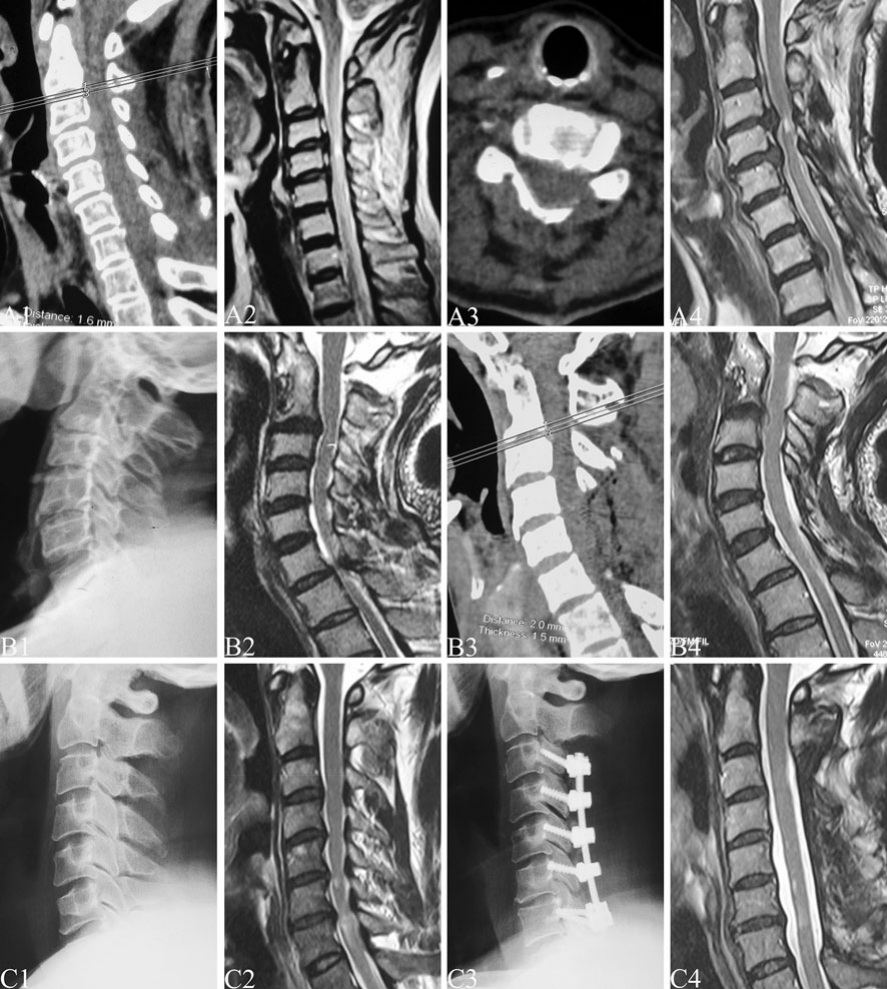
Preoperative and postoperative X-ray, CT and MRI of multilevel cervical spondylotic myelopathy treated by different posterior operations. **a** A 67-year-old female patient underwent C4 to C7 laminoplasty, whose preoperative JOA score was 8. In the final follow-up of 9.5 years, the loss of CI was 2.4 %, final follow-up JOA score was 14 and NDI score was 23. **b** A 56-year-old male patient underwent C4 to C6 laminectomy, whose preoperative JOA score was 7. In the final follow-up of 7.5 years, the loss of CI was 3.7 %, final follow-up JOA score was 13 and NDI score was 27. **c** A 61-year-old male patient underwent C3 to C7 laminectomy with lateral mass screw fixation, whose preoperative JOA score was 8. In the final follow-up of 8 years, the loss of CI was 0.4 %, final follow-up JOA score was 16 and NDI score was 2

**Fig. 3:**
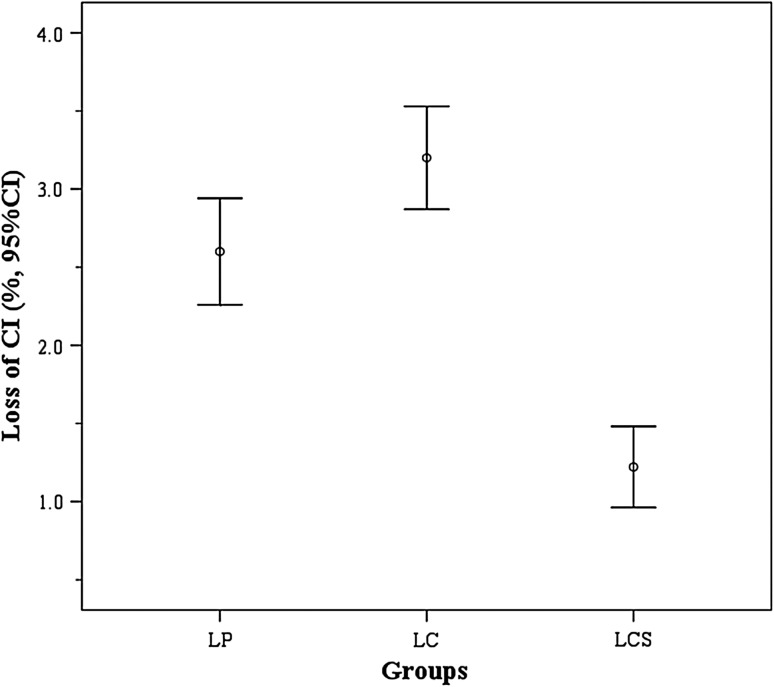
Loss of cervical curvature index in each group. The difference in the three groups for loss of CI among was statistically significant (*F* = 41.46, *P* < 0.001)

There were statistically significant differences between preoperative and final follow-up CIs in LP and LC groups (*P* < 0.05), while no significant difference between preoperative and final follow-up CI in group LCS (*t* = 0.96, *P* > 0.34). In the final follow-up, the loss of CI was 2.60 % in group LP, 3.20 % in group LC, and 1.22 % in group LCS, respectively (Table 2; Fig. 2). The difference in the three groups for loss of CI among was also statistically significant (*F* = 41.46, *P* < 0.001) (Fig. 3). The Student–Newman–Keuls test showed significant differences in loss of CI between groups LP and LCS (*P* < 0.05), between groups LC and LCS (*P* < 0.05), and between groups LP and LC (*P* < 0.05).

**Table 2:** Preoperative and final follow-up cervical curvature index in each group

Parameter	Group LP (*n* = 36)	Group LC (*n* = 30)	Group LCS (*n* = 32)	*F* value	*P* value
Preoperative CI (%)^a^	15.8 ± 4.3	16.1 ± 5.1	15.3 ± 4.7	0.23	0.79
Final follow-up CI (%)^a^	13.2 ± 4.6	12.9 ± 6.1	14.1 ± 5.3	0.43	0.65
Loss of CI (%)^b^	2.60 ± 1.01	3.20 ± 0.88	1.22 ± 0.72	41.46	<0.001
*t* value	2.48	2.20	0.96		
*P* value	0.016	0.031	0.34		

^a^ANOVA

^b^Student–Newman–Keuls test

### Functional results

In this study, the preoperative and final follow-up JOA scores were 8.1/14.0 in group LP, 8.1/13.1 in group LC, 8.2/14.3 in group LCS, respectively (Fig. 2). There were statistically significant differences between preoperative and final follow-up JOA scores in each group (*P* < 0.001) and in final follow-up JOA scores among the three groups (*F* = 7.81, *P* < 0.001). No significant differences in preoperative JOA scores among the three groups and in final follow-up JOA scores between groups LP and LCS were noted.

**Fig. 4:**
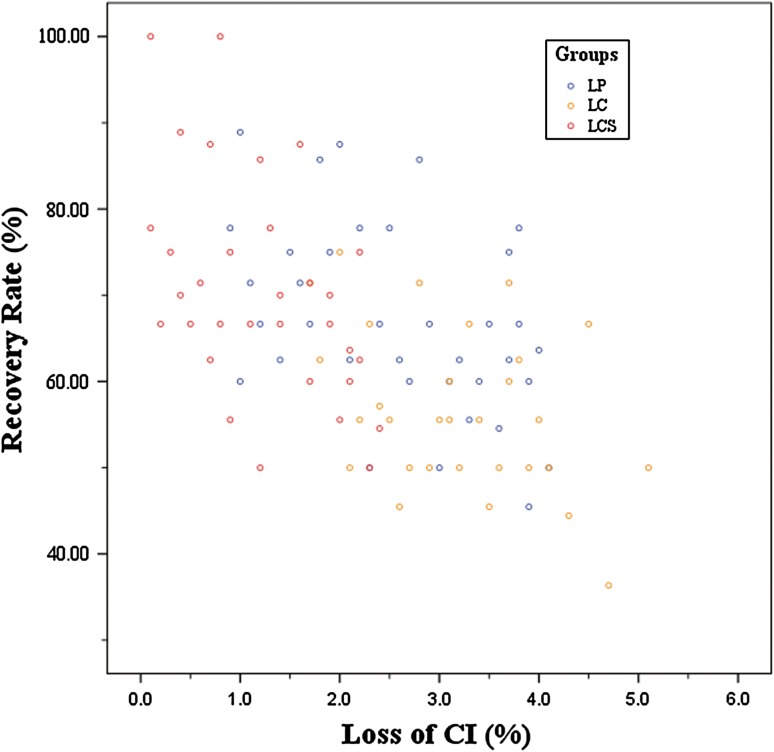
Correlation between loss of CI and recovery rate (*r* = −0.555, *P* < 0.001)

The neurological recovery were excellent in 11 (30.6 %, LP), 1 (3.3 %, LC), 11 (34.4 %, LCS) patients, good in 24 (66.7 %, LP), 25 (83.3 %, LC), 21 (65.6 %, LCS), fair in 1 (2.8 %, LP), 4 (13.3 %, LC), 0 (0 %, LCS), and there were no poor cases in three groups. The final follow-up JOA score and the improvement rate were 13.97 ± 1.28 and 66.90 % ± 11.05 %, 13.07 ± 1.23 and 56.55 % ± 9.39 %, and 14.31 ± 1.33 and 70.54 % ± 12.80 % in the final follow-up after laminoplasty, laminectomy alone and laminectomy with lateral mass screw fixation (Table 3). ANOVA showed significant differences among the three groups for recovery rate (*F* = 12.98, *P* < 0.001). The difference in neurological recovery rates between groups LP and LC (*P* < 0.05) and between groups LC and LCS (*P* < 0.05) was statistically significant, but there was no significant difference in the recovery rates of groups LP and LCS. Recovery rate showed a negative correlation with loss of curvature index (*r* = −0.555, *P* < 0.001) (Fig. 4).

**Table 3:** Preoperative, final follow-up JOA score and neurological recovery rate in each group

Parameter	Group LP (*n* = 36)	Group LC (*n* = 30)	Group LCS (*n* = 32)	Statistic value	*P* value
JOA score^a^					
Preoperative	8.08 ± 1.13	8.10 ± 1.18	8.16 ± 1.11	0.04	0.96
Final follow-up	13.97 ± 1.28	13.07 ± 1.23	14.31 ± 1.33	7.81	<0.001
Neurological recovery rate grade^b^					
Excellent (≥75 %)	11	1	11	13.58	0.0011
Good (50–74 %)	24	25	21		
Fair (25–49 %)	1	4	0		
Poor (<25 %)	0	0	0		
Recovery rate (%)^c^	66.90 ± 11.05	56.55 ± 9.39	70.54 ± 12.80	12.98	<0.001

^a^ANOVA; paired *t* test: statistically significant differences between preoperative and final follow-up values among the three groups

^b^Cochran-Mantel–Haenszel statistics (based on rank scores)

^c^ANOVA

### Axial symptoms

In the final follow-up, NDI score was 9.92 in group LP, 14.07 in group LC, and 4.97 in group LCS, respectively (Fig. 2). According to the NDI ranking system, there was no disability in 12, 7 patients, mild disability in 16, 9, moderate disability in 7, 10, severe disability in 1, 3, and complete disability in 0, 1 in the LP and LC groups, respectively. Within group LCS, the NDI ranking system indicated no disability in 20 patients, mild disability in 10, moderate disability in 2, and there were no severe and complete disability cases (Table 4).

**Table 4:** Axial symptom severity (NDI scores) in each group

Axial symptoms	Group LP (*n* = 36)	Group LC (*n* = 30)	Group LCS (*n* = 32)	Statistic value	*P* value
NDI scores^a^	9.92 (0–28)	14.07 (1–37)	4.97 (0–17)	18.04	<0.001
NDI ranking system^b^					
No disability	12 (33.3 %)	7 (23.3 %)	20 (62.5 %)	15.99	<0.001
Mild disability	16	9	10		
Moderate disability	7	10	2		
Severe disability	1	3	0		
Complete disability	0	1	0		

^a^Kruskal–Wallis nonparametric ANOVA

^b^Cochran-Mantel–Haenszel statistics (based on rank scores)

**Fig. 5:**
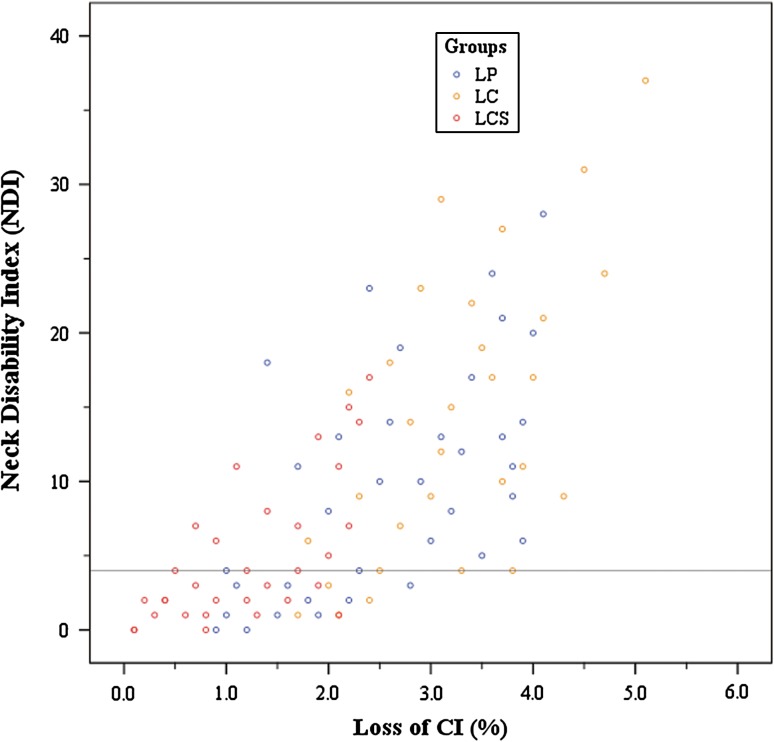
Correlation between loss of CI and axial symptoms (*r* = 0.696, *P* < 0.001)

Axial symptom incidence was 66.7 % (24/36 patients) in group LP, 76.7 % (23/30 patients) in group LC, and 37.5 % (12/32 patients) in group LCS, respectively. Kruskal–Wallis nonparametric ANOVA showed significant differences among the three groups for axial symptoms (*χ* ^2^ = 18.04, *P* < 0.001). A subsequent Bonferroni *t* test for axial symptoms showed significant differences between groups LP and LCS (*P* < 0.05), and between groups LC and LCS (*P* < 0.05), while no significant differences between groups LP and LC. Axial symptom severity was correlated with loss of curvature index (*r* = 0.696, *P* < 0.001) (Fig. 5).

## Discussion

Laminectomy is the earliest way to decompress the spinal cord by removing spinous process, lamina and ligamentum flavum in patients with multilevel cervical compressive myelopathy [3]. Laminoplasty has been performed since 1973 in Japan, and has been proved to be an effective and safe treatment to widen the spinal canal without removing the dorsal elements of the cervical spine for multisegmental CDM [1, 2]. However, some adverse outcomes of laminoplasty and laminectomy in the long-term follow-up have been reported, including significant incidences of instability, progressive kyphosis, neurological deterioration and axial symptoms [4–8]. Recently, lateral mass screws fixation has become optimal preferred option for stabilizing the cervical spine and preventing kyphotic deformity when multilevel decompression is required [9, 10]. In our institute, laminectomy and fixation were performed as an alternative to laminectomy alone in the management of multilevel CDM. During the follow-up period, we observed that some patients suffered the so-called axial symptoms including nuchal pain, neck stiffness and shoulder pain, which affect their quality of postoperative life seriously. We also conducted a literature search and found that there were few conclusive studies on this issue, particularly as to whether different posterior surgeries have long-term adverse impacts on curvature changes, neurological improvement and axial symptoms.

At final follow-up, the loss of cervical curvature was maximum in group LC, moderate in group LP, and minimum in group LCS; the differences were statistically significant among the three groups. This emphasizes the importance of early recognition of complications caused by cervical curvature changes. There is controversy on the issue: Is the loss of cervical curvature related to neurological recovery rate and axial symptoms?

In the present study, better neurological improvement was obtained in laminoplasty and laminectomy with fixation; there were statistical differences in recovery rate between groups LP and LC and between groups LC and LCS, while no significant difference between groups LP and LCS. Chiba et al. [19] followed 80 patients who underwent open-door laminoplasty for a minimum of 10 years and found that although the average JOA score and recovery rate improved significantly in 3 years after surgery, yet cervical kyphosis caused late neurological deterioration. Our results also demonstrated that cervical curvature change was correlated with neurological deterioration. Loss of cervical lordosis may be a possible factor in progressive spinal cord dysfunction [20, 21], and this issue is often discussed clinically in the pathophysiology of axial symptoms.

In patients with kyphotic deformities who underwent the laminectomy alone, the spinal cord shifted to the anterior portion of the spinal canal and abutted the posterior aspect of the vertebral bodies at the apex of the deformity. With the progression of kyphosis, the mechanical stress applied to the anterior aspect of the spinal cord eventually increased [22]. In addition, segmental instability, which is often seen at the level of kyphosis particularly in cervical flexion movement, might cause cervical degeneration acceleration and osteophytosis, thus further hindering spinal cord function recovery [23–25]. Our long-term follow-up results revealed that segmental and kyphotic instability after laminectomy could be the main cause of poor neurological recovery. Therefore, we presumed that the restoration of cervical lordosis and strengthening of cervical stability may be pivotal factors in neurological recovery.

At final follow-up, 58.2 % (57/98) of the entire group experienced axial symptoms, an incidence consistent with previous studies. The incidence of axial symptoms was 66.7 % (24/36) for group LP, 76.7 % (23/30) for group LC, and 37.5 % (12/32) for group LCS, respectively; the difference was statistically significant. The incidence of axial symptoms can be as high as 30–80 % [26], but the exact reason is unknown. Takeuchi et al. [27] believed that the axial symptoms were related to cervical kyphotic deformity. Otani et al. [28] proposed that lateral retraction of paravertebral muscles attached on the cervical spine and removal of lamina and ligamentum flavum in laminectomy, especially the semispinalis attached on the C2 spinous process, increased the flexion mechanical stress, which may be a significant factor in the development of axial symptoms. Tang et al. [29] demonstrated that the severity of neck pain and disability increases with positive sagittal malalignment following surgical reconstruction. The present study showed that axial symptom severity was positively correlated with loss of CI, which meant that the symptoms would get worse if the cervical curvature index was more severely lost. However, some patients in the final follow-up did not complain of neck pain (group LP 12/36; group LC 7/30; group LCS 20/32). Although the patients of three groups complained more or less of neck pain after surgery in short time, neck pain was gradually improved in groups LP and LCS with the restoration of cervical lordosis and strengthening of cervical stability. Our study suggested that the incidence of axial symptoms can be reduced by the restoration of cervical lordosis and strengthening of cervical stability.

Takeuchi et al. [30] and Zhang et al. [31] demonstrated that the C7 spinous process might play an important role in preventing axial symptoms, and axial symptom severity might be affected by musculature atrophy. Axial symptoms might also be caused by other problems, such as nuchal muscle intraoperative injury, destruction of facet joints, intraoperative nerve root damage and hinge side nonunion. In short, axial symptoms were the results of a complication induced by multifactor and multimechanism after posterior cervical surgery, and the explicit pathogenesis remains to be further investigated.

Successful treatment of multilevel CDM requires adequate decompression, restoration of the normal curvature and reconstruction of the cervical stability. Duan et al. [32] stated that the posterior fixations could provide immediate stability of the cervical spine following laminectomy by reinforcing the posterior tension-band, which attempted to prevent the loss of cervical lordosis and promote early neurological recovery. Ohnari et al. [26] pointed out that the reconstruction of posterior elements at laminoplasty was expected to relieve axial symptoms. There were ample biomechanical experiments [9, 33] suggesting that lateral mass screws could provide rigid fixation to the multiple cervical planes: flexion stability increased 92 %, extension stability increased 60 % and rotation stability also improved greatly. During the follow-up period, based on our observations and experience [9, 10, 34, 35], we have favored laminectomy with lateral mass screw fixation to perform adequate decompression of the spinal cord and maintain normal cervical alignment, which has obtained excellent neurological improvement and minimal incidence of complications.

This study has some limitations. Over the last decade, many modified anterior or posterior surgical approaches for the treatment of multilevel CDM, including multilevel anterior cervical discectomy with fusion (ACDF), noncontiguous anterior decompression and fusion (NADF), oblique cervical corpectomy (OCC), and combined anterior-posterior fusion, had been developed and obtained favorable outcomes [36–40]. However, some surgical methods in this study, e.g. LC, had been rarely used in recent years owing to a high incidence of the long-term surgery-related complications. In the present study, we only selected the patients from our medical center, and all surgeries were performed by the same surgical team. There is still a need for prospective, large-scale, multi-center clinical trials to further confirm our results.

## Conclusions

Better neurological improvement was obtained by laminoplasty and laminectomy with lateral mass screw fixation during the surgical management of multilevel CDM. Meanwhile, we observed that loss of cervical alignment in laminoplasty and laminectomy caused a high incidence of axial symptoms. The results show that loss of cervical lordosis is negatively associated with neurological recovery and positively related to axial symptom severity. Lateral mass screw fixation might play an important role in preventing loss of postoperative cervical curvature and decreasing the incidence of axial symptoms.

## References

[1] Derenda M, Kowalina I (2006) Cervical laminoplasty–review of surgical techniques, indications, methods of efficacy evaluation, and complications. Neurol Neurochir Pol 40:422–432; discussion 433 17103356

[2] Hale JJ, Gruson KI, Spivak JM (2006). Laminoplasty: a review of its role in compressive cervical myelopathy. Spine J.

[3] Ryken TC, Heary RF, Matz PG (2009). Cervical laminectomy for the treatment of cervical degenerative myelopathy. J Neurosurg Spine.

[4] Kaptain GJ, Simmons NE, Replogle RE, Pobereskin L (2000). Incidence and outcome of kyphotic deformity following laminectomy for cervical spondylotic myelopathy. J Neurosurg Spine.

[5] Hansen-Schwartz J, Kruse-Larsen C, Nielsen CJ (2003). Follow-up after cervical laminectomy, with special reference to instability and deformity. Br J Neurosurg.

[6] Kang SH, Rhim SC, Roh SW, Jeon SR, Baek HC (2007). Postlaminoplasty cervical range of motion: early results. J Neurosurg Spine.

[7] Hyun SJ, Rhim SC, Roh SW, Kang SH, Riew KD (2009). The time course of range of motion loss after cervical laminoplasty: a prospective study with minimum two-year follow-up. Spine.

[8] Nurboja B, Kachramanoglou C, Choi D (2012). Cervical laminectomy vs. laminoplasty: is there a difference in outcome and postoperative pain?. Neurosurgery.

[9] Houten JK, Cooper PR (2003). Laminectomy and posterior cervical plating for multilevel cervical spondylotic myelopathy and ossification of the posterior longitudinal ligament: effects on cervical alignment, spinal cord compression, and neurological outcome. Neurosurgery.

[10] Anderson PA, Matz PG, Groff MW (2009). Laminectomy and fusion for the treatment of cervical degenerative myelopathy. J Neurosurg Spine.

[11] Itoh T, Tsuji H (1985). Technical improvements and results of laminoplasty for compressive myelopathy in the cervical spine. Spine.

[12] Itoh T (1986). Clinical studies on the significance of en bloc laminoplasty for cervical compressive myelopathy. Nihon Seikeigeka Gakkai Zasshi.

[13] Pal D, Bayley E, Magaji SA, Boszczyk BM (2011). Freehand determination of the trajectory angle for cervical lateral mass screws: how accurate is it?. Eur Spine J.

[14] Takeshita K, Murakami M, Kobayashi A, Nakamura C (2001). Relationship between cervical curvature index (Ishihara) and cervical spine angle (C2–7). J Orthop Sci.

[15] Hirabayashi K, Miyakawa J, Satomi K, Maruyama T, Wakano K (1981). Operative results and postoperative progression of ossification among patients with ossification of cervical posterior longitudinal ligament. Spine.

[16] Vernon H, Mior S (1991). The Neck Disability Index: a study of reliability and validity. J Manipulative Physiol Ther.

[17] McCarthy MJ, Grevitt MP, Silcocks P, Hobbs G (2007). The reliability of the Vernon and Mior neck disability index, and its validity compared with the short form-36 health survey questionnaire. Eur Spine J.

[18] Vernon H (2008). The Neck Disability Index: state-of-the-art, 1991–2008. J Manipulative Physiol Ther.

[19] Chiba K, Ogawa Y, Ishii K (2006). Long-term results of expansive open-door laminoplasty for cervical myelopathy: average 14-year follow-up study. Spine.

[20] Stulík J, Nesnídal P, Sebesta P, Vyskočil T, Kryl J (2011). Kyphotic deformities of the cervical spine. Acta Chir Orthop Traumatol Cech.

[21] Chen Y, Guo Y, Lu X (2011). Surgical strategy for multilevel severe ossification of posterior longitudinal ligament in the cervical spine. J Spinal Disord Tech.

[22] Uchida K, Nakajima H, Sato R (2009). Cervical spondylotic myelopathy associated with kyphosis or sagittal sigmoid alignment: outcome after anterior or posterior decompression. J Neurosurg Spine.

[23] Baptiste DC, Fehlings MG (2006). Pathophysiology of cervical myelopathy. Spine J.

[24] Kuwazawa Y, Bashir W, Pope MH, Takahashi K, Smith FW (2006). Biomechanical aspects of the cervical cord: effects of postural changes in healthy volunteers using positional magnetic resonance imaging. J Spinal Disord Tech.

[25] Wang B, Liu H, Wang H, Zhou D (2006). Segmental instability in cervical spondylotic myelopathy with severe disc degeneration. Spine.

[26] Ohnari H, Sasai K, Akagi S, Iida H, Takanori S, Kato I (2006). Investigation of axial symptoms after cervical laminoplasty, using questionnaire survey. Spine J.

[27] Takeuchi K, Yokoyama T, Aburakawa S (2005). Axial symptoms after cervical laminoplasty with C3 laminectomy compared with conventional C3–C7 laminoplasty: a modified laminoplasty preserving the semispinalis cervicis inserted into axis. Spine.

[28] Otani K, Sato K, Yabuki S, Iwabuchi M, Kikuchi S (2009). A segmental partial laminectomy for cervical spondylotic myelopathy: anatomical basis and clinical outcome in comparison with expansive open-door laminoplasty. Spine.

[29] Tang JA, Scheer JK, Smith JS (2012). The Impact of Standing Regional Cervical Sagittal Alignment on Outcomes in Posterior Cervical Fusion Surgery. Neurosurgery.

[30] Takeuchi T, Shono Y (2007). Importance of preserving the C7 spinous process and attached nuchal ligament in French-door laminoplasty to reduce postoperative axial symptoms. Eur Spine J.

[31] Zhang P, Shen Y, Zhang YZ, Ding WY, Xu JX, Cao JM (2011). Preserving the C7 spinous process in laminectomy combined with lateral mass screw to prevent axial symptom. J Orthop Sci.

[32] Duan Y, Zhang H, Min SX, Zhang L, Jin AM (2011). Posterior cervical fixation following laminectomy: a stress analysis of three techniques. Eur Spine J.

[33] Singh K, Vaccaro AR, Kim J, Lorenz EP, Lim TH, An HS (2003). Biomechanical comparison of cervical spine reconstructive techniques after a multilevel corpectomy of the cervical spine. Spine.

[34] Audat ZA, Barbarawi MM, Obeidat MM (2011). Posterior cervical decompressive laminectomy and lateral mass screw fixation. Neurosciences (Riyadh).

[35] Aydogan M, Enercan M, Hamzaoglu A, Alanay A (2012). Reconstruction of the subaxial cervical spine using lateral mass and facet screw instrumentation. Spine.

[36] Lin Q, Zhou X, Wang X (2012). A comparison of anterior cervical discectomy and corpectomy in patients with multilevel cervical spondylotic myelopathy. Eur Spine J.

[37] Chacko AG, Joseph M, Turel MK (2012). Multilevel oblique corpectomy for cervical spondylotic myelopathy preserves segmental motion. Eur Spine J.

[38] Liu T, Xu W, Cheng T (2011). Anterior versus posterior surgery for multilevel cervical myelopathy, which one is better? A systematic review. Eur Spine J.

[39] Lian XF, Xu JG, Zeng BF (2010). Noncontiguous anterior decompression and fusion for multilevel cervical spondylotic myelopathy: a prospective randomized control clinical study. Eur Spine J.

[40] Koller H, Schmidt R, Mayer M (2010). The stabilizing potential of anterior, posterior and combined techniques for the reconstruction of a 2-level cervical corpectomy model: biomechanical study and first results of ATPS prototyping. Eur Spine J.

